# PD29 Using Different Parametric Distributions In Partitioned Survival Analysis: Impact On Incremental Cost-Utility Ratio In A Proportional Hazards Model

**DOI:** 10.1017/S0266462324002927

**Published:** 2025-01-07

**Authors:** Ludmila Gargano, Layssa Oliveira, Haliton Alves De Oliveira Junior, Rosa Lucchetta

## Abstract

**Introduction:**

Partitioned survival analysis (PtSA) is a useful modeling technique, especially in oncology, in which different parametric distributions can be used to extrapolate survival data. Many studies lack justification for their chosen distributions, neglecting exploration of uncertainty in extrapolated estimates. We evaluated how different distributions impact the incremental cost-utility ratio (ICUR) for docetaxel, compared with abiraterone plus docetaxel (AA+DTX), for prostate cancer.

**Methods:**

A three-state PtSA was constructed using overall survival (OS) and progression-free survival (PFS) curves from a docetaxel trial extrapolated over a 20-year horizon with exponential, Weibull, and Gompertz distributions. Log-normal, log-logistic, and generalized gamma were not considered because they are not compatible with the proportional hazards assumption. Curves for AA+DTX were adjusted using hazard ratios (HRs) from indirect comparisons for OS (HR 0.75, 95% confidence interval [CI]: 0.59, 0.95) and PFS (HR 0.50, 95% CI: 0.35, 0.72). Assessment included visual inspection, clinical plausibility, and Akaike and Bayesian information criterion (AIC/BIC) statistics. Utility values, medication costs, and disease monitoring costs were considered based on health state (pre- or post-progression).

**Results:**

For docetaxel PFS, visual inspection showed no significant differences between the three distributions used for the proportional hazards model, while the exponential model showed the longest tail for OS. When choosing by the lowest AIC/BIC (OS: Weibull; PFS: exponential), cost-effectiveness analysis resulted in an ICUR of BRL79,224 (USD16,139) per quality-adjusted life-year (QALY). The maximum and minimum ICUR was BRL81,559 (USD16,615) (OS: Gompertz; PFS: Weibull) and BRL70,136 (USD14,288) (OS: exponential; PFS: Weibull), respectively, which represented an important variation in the base case scenario (AIC/BIC) ICUR.

**Conclusions:**

Using the Brazilian cost-effectiveness threshold (BRL120,000 [USD24,446] per QALY gained) and choosing any of the three distributions of the proportional hazards model, AA+DTX would be considered cost effective, which would not change the direction of the recommendation. However, despite the few recommendations in the literature regarding the adoption of parametric models for economic analyses in health, it is important to explore scenarios with different distributions.

